# A long way from Laos

**DOI:** 10.1371/journal.pntd.0006534

**Published:** 2018-08-30

**Authors:** Jade Ramos-Poblete, Erica Kasper, Anandit Mu

**Affiliations:** Division of Infectious Diseases, University of California San Francisco, Fresno Medical Education Program, Fresno, California, United States of America; S. Cuore Hospital, ITALY

A 50-year-old male emigrated from Laos to California in the 1980s. Thirty years later, he was diagnosed with nephrotic range proteinuria due to membranoproliferative glomerulonephritis. In addition to medications for gout, anemia, hypertension, and a pulmonary embolism, he was started on prednisone (60 mg daily) and mycophenolate mofetil (1000 mg twice daily). Three months later, he presented with melena, weakness, left upper quadrant abdominal pain, fever, and hypotension. His blood pressure was 80/57 mmHg. Laboratory workup revealed a hemoglobin of 4.2 mg/dL, a mean corpuscular volume of 76, and a white blood cell count of 11.8 × 10^9^ cells/L without eosinophilia. His renal function was at baseline, with the rest of the blood chemistry, including liver enzymes, within normal limits. Computed tomography of the abdomen was unremarkable. Esophagoduodenoscopy and colonoscopy demonstrated mild esophagitis, patchy ileitis, and ulcers in the gastric atrum, splenic flexure, descending colon, sigmoid colon, and rectosigmoid area. Histopathology of duodenal biopsies is shown in Figs 1 and 2.

What is your diagnosis?

**Fig 1:**
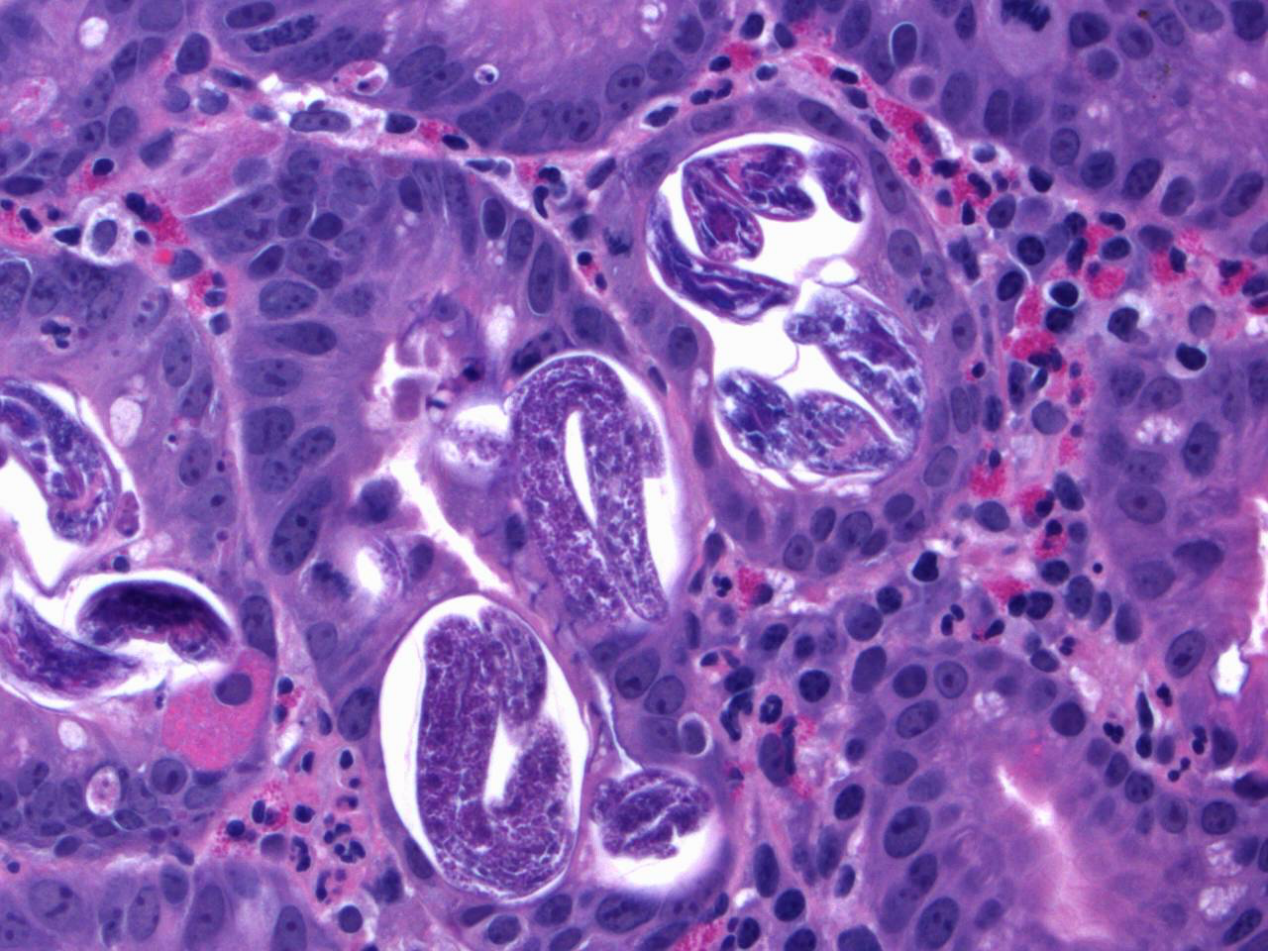
Histopathology of duodenal biopsy (hematoxylin and eosin staining, original magnification × 400).

**Fig 2:**
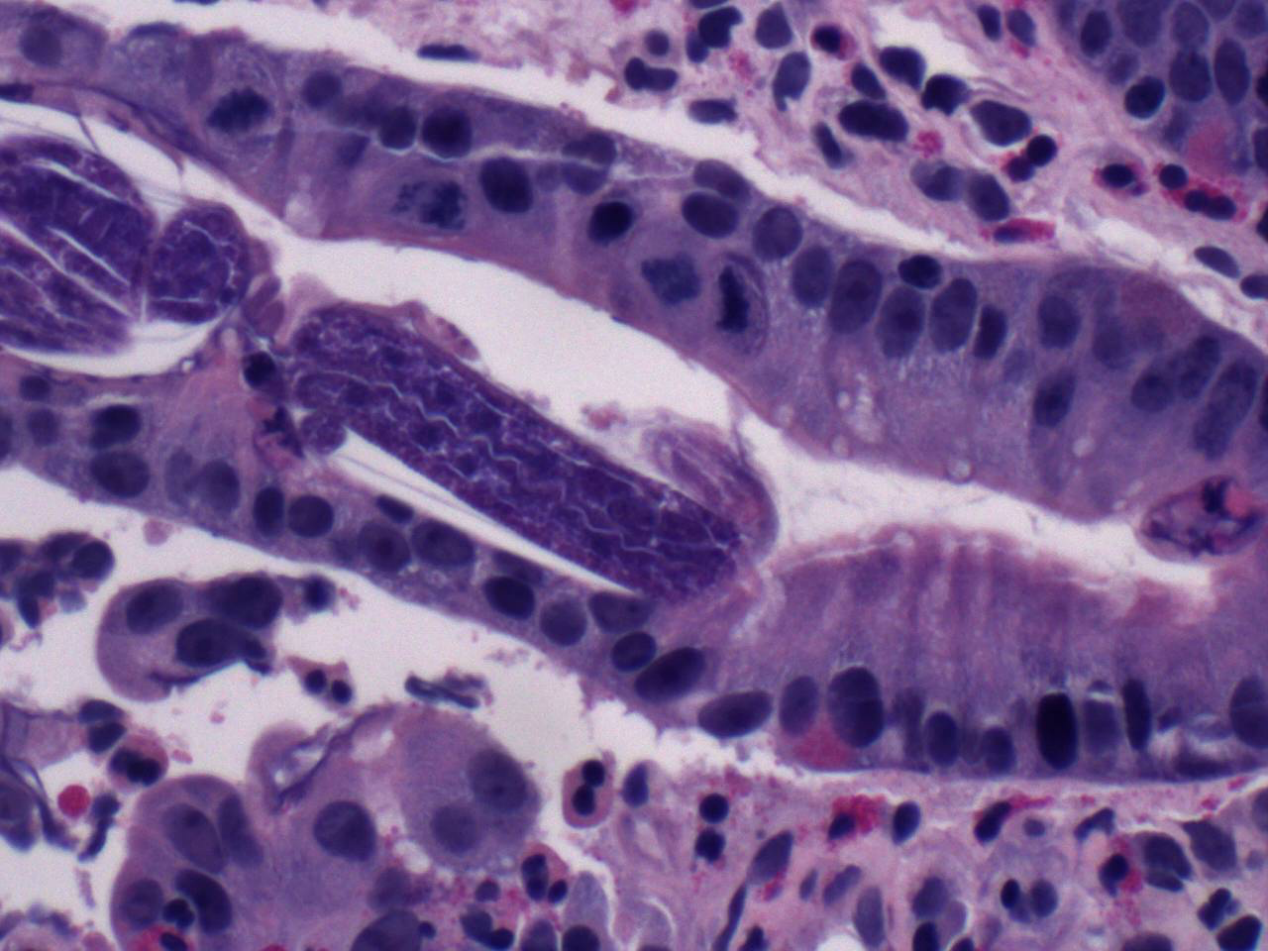
Duodenal biopsy (hematoxylin and eosin stain, 400× magnification).

## Diagnosis

### *Strongyloides stercoralis* hyperinfection

The duodenal biopsy showed patchy mild chronic inflammation, villous edema, villous blunting, and parasites within crypts, consistent with *Strongyloides stercoralis* infection. Two stool specimens were negative for ova and parasites by trichrome stain; however, *Strongyloides* serology (IgG) was positive by enzyme-linked immunosorbent assay (ELISA, Quest Diagnostics, Valencia, California). Human immunodeficiency virus (HIV) antibody screening was negative, as was polymerase chain reaction for human T-cell lymphotropic virus (HTLV) I and II DNA. The patient received seven days of oral ivermectin (200 μg/kg daily).

Chronic strongyloidiasis can be asymptomatic or manifest with minimal gastrointestinal or cutaneous symptoms in people living in endemic areas. The decades-long infection begins with filariform larvae of *Strongyloides* penetrating intact skin. They are transported hematogenously to the lungs, penetrating alveolar spaces. Ascending to the trachea and pharynx, they are swallowed and transported to the small intestine, where larvae become adult female worms producing eggs, which yield rhabditiform larvae. Rhabditiform larvae may be passed in the stool or cause autoinfection of the host by transforming to infective filariform larvae, which can penetrate intestinal mucosa or the skin of the perianal area, once again [1,2]. Thus, the life cycle of the nematode is complete within the human host and may coexist without detection in endemic regions such as Laos, where reported prevalence ranges from 4%–41% [3–5].

Migrants seen at GeoSentinel clinics in all regions of the world have had strongyloidiasis at a rate of about 5% [6]. Detection of larvae remains the gold standard for confirming the diagnosis but has low sensitivity; obtaining three or more successive stool samples for ova and parasites is recommended, as a single stool examination fails to detect larvae in up to 70% of cases. While serologic testing is useful, antibodies can cross react with other nematode infections and do not distinguish between recent and previously treated infections [7]. Real-time polymerase chain reaction (RT-PCR) is a promising diagnostic method that detects *Strongyloides* DNA in stool samples as a confirmatory method to follow serologic screening in nonendemic areas [8].

Hyperinfection is accelerated by immunosuppression, be it corticosteroids, HIV, HTLV-1, hematologic malignancy, or organ transplantation. Any subject coming from an endemic area must be screened for *Strongyloides*, at least with serology, before starting any immune suppressant and particularly corticosteroids [9–11]. Gastrointestinal mucosal ulceration may lead to massive hemorrhage and resultant mortality rates as high as 50%–70% [10,12]. Endoscopic biopsy reveals parasites in the gastric crypts or duodenal glands with eosinophilic infiltration of the lamina propria. Ivermectin is the first-line treatment, with a recommended dose of 200 μg/kg orally for one to two days for acute and chronic strongyloidiasis, but longer courses are needed in hyperinfection in the setting of immunosuppression [12–15].

Our patient recovered from strongyloidiasis, but his health was subsequently affected by reactivation of hepatitis B virus, multidermatomal varicella zoster, and disseminated nocardiosis with cutaneous, pancreatic, pulmonary, cerebral, and cerebellar involvement. Remarkably, he recovered from these infections as well and is now maintained on hemodialysis for end-stage renal disease.

**Key teaching points**

- Chronic strongyloidiasis can persist asymptomatically for many years, even for life, when a population of adult worms is maintained in the small intestine of people living in endemic areas.
- In immunosuppressed individuals, severe, disseminated strongyloidiasis can occur as infective larvae invade the tissues of the duodenum and jejunum and circulate throughout the body, causing widespread organ damage. Individuals from endemic areas must be screened for strongyloides with serology prior to commencing immune suppression.
- Ivermectin is highly efficacious and is the drug of choice for therapy. Parasitological cure is similar with thiabendazole, but its use is limited by toxicities and adverse effects.
